# Biophysical insights into the molecular mechanisms of beta amyloid aggregation and its toxic effects in Alzheimer’s disease

**DOI:** 10.3389/fmolb.2025.1704653

**Published:** 2025-10-23

**Authors:** Soghra Bagheri, Luciano Saso

**Affiliations:** ^1^ Medical Biology Research Center, Health Technology Institute, Kermanshah University of Medical Sciences, Kermanshah, Iran; ^2^ Department of Physiology and Pharmacology “Vittorio Erspamer”, Sapienza University, Rome, Italy

**Keywords:** Alzheimer’s disease, amyloid aggregation, molecular mechanisms, membranes, damage, copper, cholesterol, oxidized

## Abstract

Alzheimer’s disease is recognized as the most common neurodegenerative disorder, characterized by the presence of amyloid plaques, which have consistently garnered significant attention. Since the disease was first identified, extensive research has been devoted to investigating these plaques. As our understanding of the disease has progressed, the detrimental role of plaques has been questioned, leading to the hypothesis that amyloid oligomeric aggregates are the main culprits. Nevertheless, subsequent research indicated that the concentrations of amyloids employed in the experiments were considerably elevated compared to physiological conditions, and that at physiological concentrations, amyloids do not exhibit significant accumulation or toxicity. This article aims to offer a detailed biophysical perspective on the formation of amyloid aggregates under physiological conditions and their impact on membranes, providing valuable insights for researchers in this field.

## 1 Introduction

Amyloid plaques, primarily composed of amyloid beta (Aβ) peptides, are a key feature of Alzheimer’s disease (AD). The detection of Aβ plaques in individuals without the disease prompted the consideration of alternative theories concerning the fundamental cause of AD, such as the cholinergic hypothesis, Aβ oligomer hypothesis, tau hypothesis, mitochondrial cascade hypothesis, calcium homeostasis hypothesis, neurovascular hypothesis, inflammatory hypothesis, metal ion hypothesis, lymphatic system hypothesis, microbial hypothesis, and others (Bagheri et al., 2022; Bagheri et al., 2024; Liu et al., 2019). One of these theories is the “amyloid toxic oligomers hypothesis,” which posits that small, soluble Aβ oligomers are the main source of toxicity, rather than the amyloid plaques themselves (Bagheri et al., 2018). The mechanisms through which Aβ oligomers contribute to the neuropathogenesis associated with AD progression encompass receptor interaction, disruption of cell membranes, impairment of mitochondrial function, dysregulation of calcium homeostasis, and the induction of tau pathology (Huang and Liu, 2020). The effectiveness of medications such as EPPS (Zarrilli et al., 2024) or 8-Hydroxyquinolines (8-HQs) (Ryan et al., 2015), which inhibit the formation of neurotoxic Aβ oligomers by stabilizing smaller, non-toxic aggregates, lends support to the toxic oligomer hypothesis.

Given that Aβ peptides do not typically accumulate under healthy condition and perform numerous physiological functions (Bishop and Robinson, 2004), extensive research has been conducted to understand the reasons and mechanisms behind their accumulation and to answer the question of whether Aβ peptide accumulation is truly the main cause of AD (Bagheri et al., 2018). In the majority of *in vitro* experiments, the Aβ concentration needed is significantly greater than what is typically found in physiological conditions, and no accumulation is detected within the physiological concentration range of Aβ (Lyubchenko, 2020). This article delves into the molecular mechanisms contributing to Aβ accumulation from a biophysical perspective, taking into account the latest scientific advancements.

## 2 Aβ peptides

Aβ is produced via the proteolytic cleavage of the transmembrane protein amyloid precursor protein (APP), a process facilitated by the enzymes β-secretase and γ-secretase (Chen et al., 2017). β-secretase can cleave APP at each of the first 11 residues of the β-site, resulting in the formation of secreted-APP-β and a membrane-associated fragment. Subsequently, γ-secretase further cleaves membrane-associated fragment at various sites, leading to the production of Aβ peptides of varying lengths. The predominant Aβ peptides generated are 40 amino acids in length; however, peptides with lengths ranging from 38 to 43, and even 46 and 49 amino acids, have been identified *in vivo* (Zhang et al., 2012). The two predominant alloforms of the peptide are present simultaneously under physiological conditions in the brain, exhibiting an Aβ42:Aβ40 ratio of approximately 1:9. In AD patients, this ratio frequently shifts to a greater proportion of Aβ42, which has a greater tendency to aggregate (Gu and Guo, 2013; Pauwels et al., 2012).

### 2.1 Aβ40

Nuclear magnetic resonance (NMR) diffusion studies show that at 37 °C, soluble Aβ40 retains its disordered structure and predominantly exists as a monomer, although dimers and other small oligomeric configurations have been observed (Leite et al., 2020). Different oligomer species are in rapid equilibrium, with intensity decreasing as the oligomer size increases (Bitan et al., 2003; Pujol-Pina et al., 2015). Aβ40 dimers and trimers adopt a globular shape without a well-defined secondary structure (Pujol-Pina et al., 2015).

A study using native ion mobility mass spectrometry and molecular dynamics (MD) simulations discovered that a specific β-hairpin motif in the Aβ40 peptide sequence is essential for the formation of Aβ oligomers. Initially, the oligomers grow in a spherical manner, but as they exceed tetramers in size, they start to form extended linear aggregates (Khaled et al., 2023).

**FIGURE 1:**
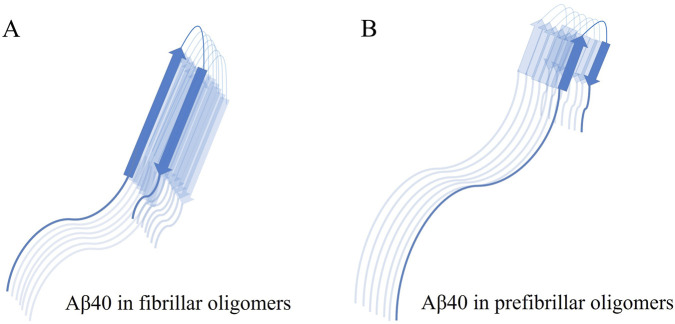
Conformation of Aβ40 monomer in different classes of Aβ40 oligomers. Fibrillar oligomers are characterized by their high β-sheet content and structural similarities to fibrils **(A)**. In contrast, prefibrillar oligomers lack a significant amount of β-sheets and mainly display a disordered structure, with irregularities in residues 1 to 25, while residues 26 to 40 form an antiparallel β-barrel **(B)**.

Conformation-dependent antibodies suggest the existence of at least two fundamentally different categories of amyloid oligomers: fibrillar (FO) and prefibrillar oligomers (PFO) (Glabe, 2008). CD, FTIR, and Raman spectroscopic analyses show that PFOs lack a significant proportion of β-sheets and largely display a disordered conformation. The denaturation process of PFOs occurs at low concentrations of guanidinium thiocyanate with low cooperativity, suggesting weak intermolecular interactions. A proposed structural model of PFOs suggests that residues 1–25 are disordered, while residues 26–40 are organized into an antiparallel β-barrel (Figure 1). In contrast, Aβ40 FOs are small aggregates characterized by a high content of β-sheets and exhibit structural similarities to fibrils, albeit with some irregularities in the stacking of β-sheets. However, they demonstrate reduced stability when subjected to denaturation (Breydo et al., 2016).

In various oligomeric preparations of numerous amyloidogenic proteins and peptides, annular protofibrils (APFs) have been characterized as ring-shaped or pore-like structures. Their significance lies in the fact that their pore-like structure aligns with multiple studies indicating the membrane-permeabilizing capabilities of amyloid oligomers. It seems that APFs are generated through the circularization of PFO subunits (Kayed et al., 2009).

Experimental observations indicate that high molecular weight oligomers of Aβ40 (Rh ∼ 20 nm) transform into more compact fibril nuclei (Rh ∼ 10 nm), while additional fibril nuclei are generated through processes catalyzed by the fibril surface, subsequently leading to the growth of fibrils through the incorporation of soluble Aβ species (Leite et al., 2020). The transient association of Aβ40 monomers with fibers reveals partially bound conformations, with the most significant interactions observed around the F19-K28 region, while interactions near the C-terminus (L34-G37) are relatively weaker (Brender et al., 2019). Previous research has shown that the gradual interchange between Aβ40 monomers and fibrils correlates with dynamic interactions occurring at the C-terminus (Kotler, 2015).

A notable characteristic shared by the various fibril structures identified for Aβ40 is the existence of two β-sheets. The first, known as the N-sheet, includes residues Q15-D23, while the second, referred to as the C-sheet, is composed of residues A30-V36 (Kunnath Muhammedkutty and Zhou, 2025). The predominant polymorph of brain-derived Aβ40 fibrils found in typical AD patients exhibits completely extended conformations for the molecules in the central two cross-β layers, with β-hairpin conformations observed for the molecules in the outer cross-β layers (Ghosh et al., 2021). The intermolecular interaction between Gly25 and Ile31 prevents the typical hairpin configuration seen in Aβ40 fibrils, promoting the extended conformation in these specific monomers (Scheidt et al., 2024).

### 2.2 Aβ42

Aβ42 monomers consist of various ordered and disordered conformational species (Zhuang et al., 2010). Although Aβ42 and Aβ40 share the same amino acid sequence with the exception of two extra C-terminal residues, Aβ42 exhibits a greater tendency to aggregate compared to Aβ40 (Mamone et al., 2021). The C-terminus of Aβ42 has greater structural integrity compared to Aβ40, with the development of a β-hairpin within the sequence 31–42, leading to decreased flexibility and increased fibril formation (Sgourakis et al., 2007). The free energy landscape of amyloid nucleation shows that the C-terminus (residues 29–42) of Aβ42 is crucial in the rate-limiting step of nucleation (Arutyunyan et al., 2025). The dynamic behavior of the C-terminal residues in Aβ42 affects the backbone dynamics of residues V24 to K28 (Mamone et al., 2021). The Aβ42 monomer often assumes conformations in which the N-terminus region is structured in a manner closely resembling its configuration within the fibril. This inherent tendency of the monomeric Aβ to take on fibril-like conformations may account for the minimal free energy barrier associated with the elongation of Aβ42 fibrils (Barz et al., 2021). Findings from *in situ* atomic force microscopy (AFM) show that the typical growth rates of Aβ42 fibrils are approximately 50 times quicker than those of Aβ40 fibrils (Sunil Menon et al., 2025).

Electrospray ionization ion mobility mass spectrometry (ESI-IM-MS) analysis reveals that Aβ42 primarily oligomerizes into dimers and trimers, which take on a spherical form and do not exhibit a clear secondary structure (Pujol-Pina et al., 2015). ESI-IMS-MS is an analytical technique that is advancing swiftly for the analysis of intricate compounds in the gas phase. ESI-IMS-MS effectively separates isomers, offers structural insights, and quantitatively identifies peptides, lipids, carbohydrates, polymers, and metabolites found in biological samples (Kohoutek and Harrington, 2023). Transmission electron microscopy and atomic force microscopy (AFM) data analysis showed that round particles, averaging 10–15 nm in width, were more toxic than fibrils and protofibrils. Size exclusion chromatography indicated a single size distribution of 24 ± 3 kDa for the oligomer samples, suggesting a pentameric structure. These oligomers lack the β-sheet structure present in fibrils and instead consist of loosely aggregated strands with protected C termini and a turn conformation, facilitating interaction between Phe19 and Leu34 (Ahmed et al., 2010). Unlike fibrils, the β-strands in oligomers are not arranged in in-register parallel β-sheets. This unique arrangement of β-strands may act as an energy barrier, impeding the transformation of oligomers into fibrils or the initiation of monomer conversion into fibrils (Tay et al., 2013).

The 3D structure of a disease-associated Aβ42 fibril polymorph, determined through the integration of solid-state NMR spectroscopy data and mass-per-length assessments from electron microscopy, reveals the presence of two molecules within each fibril layer. This structure is defined by residues 15–42, forming a double-horseshoe-like cross-β-sheet configuration that effectively hides hydrophobic side chains. In contrast, residues 1–14 show partial ordering and adopt a β-strand conformation (Wälti et al., 2016). Studies on Aβ42 fibrils, whether synthesized *in vitro* or extracted from brain tissue, have identified polymorphs with variations in amino acid side chain orientations, structurally ordered segment lengths, and interactions between cross-β subunit pairs within a single filament, while maintaining a common S-shaped configuration for individual Aβ42 molecules. In contrast, fibrils generated through seed growth, using tissue fibrils as seeds, exhibit distinct structural features. These structures qualitatively differ in various aspects, displaying molecular configurations resembling the Greek letters ν and υ rather than the letter S (Lee et al., 2023).

The application of high-speed AFM technique revealed two distinct morphomers, “straight” and “spiral,” formed during Aβ42 fibril aggregation. Each growth mode is contingent upon the structure of the initial fibril nucleus; however, transitions from one growth mode to another are sometimes detected. This indicates that the structure at the end of the fibril oscillates between the two growth modes and is indeed influenced by relatively minor alterations in environmental conditions (Watanabe-Nakayama et al., 2016). *In situ* AFM imaging identified two distinct aggregation pathways that do not cross: one leading to amyloid fibrils and the other to oligomers and amorphous aggregates (Sunil Menon et al., 2025). ThT analysis and high-speed AFM observations showed that globular-shaped Aβ oligomers (gAβO) facilitated the fibril formation of low molecular weight Aβ42 (including dimers, trimers, and oligomers with fewer than 8 monomers), whereas gAβO itself did not lead to the formation of fibrous aggregates (Nakano et al., 2025).

## 3 Amyloid content of plaques

In the brain of patients with sporadic AD (SAD), amyloid plaques are mainly composed of Aβ42, with some plaques containing only Aβ42, despite the higher concentration of Aβ40 (Gu and Guo, 2013; Tehrani et al., 2024). Previous studies have shown that diffuse plaques in SAD brains are exclusively positive for Aβ42 and negative for Aβ40. In contrast, in the cortices of elderly people not living with dementia, senile plaques often exhibit strong positivity for Aβ40 (Iwatsubo et al., 1994).

Analysis of cortical specimens from familial AD (FAD) patients with the APP717 (Val to Ile) mutation revealed a high prevalence of plaques positive for Aβ42 and negative for Aβ40 (Iwatsubo et al., 1994). In contrast, FAD patients with the Arctic (E22G) mutation in Aβ exhibit a higher Aβ40 concentration in the core of amyloid plaques. Cotton-wool plaques, large spherical structures up to approximately 120 μm in diameter, larger than the typical 10 μm diameter of amyloid plaques in sporadic AD, are more common in Arctic FAD cases (Tehrani et al., 2024). High-resolution cryo-electron microscopy analysis of Aβ filaments extracted from the frontal cortex of an individual with the Arctic mutation revealed that the predominant filaments consist of two distinct pairs of protofilaments: residues V12-V40 (human Arctic fold A) and E11-G37 (human Arctic fold B) (Yang et al., 2023).

Reduced levels of insoluble Aβ42 were observed in the frontal and temporal cortex of APPsw mutation carriers. In contrast, presenilin-1 (PS1) mutation carriers showed significantly lower levels of both insoluble Aβ40 and Aβ42 in all four cortical regions compared to those with SAD. Additionally, individuals with the PS1 mutation exhibited a notably elevated ratio of insoluble Aβ42 to Aβ40 (Hellström-Lindahl et al., 2009). PS1 acts as the catalytic component of γ-secretase, an intramembranous protease responsible for cleaving APP. Notably, mutations in the PSEN1 gene, encoding PS1, represent the predominant cause of FAD (Kelleher and Shen, 2017).

## 4 Differences between Aβ40 and Aβ42 in gathering

The aggregate-free Aβ40 exists in the forms of monomers, dimers, trimers, and tetramers, maintaining a rapid equilibrium state. In contrast, Aβ42 tends to preferentially form pentamer and hexamer units, known as paranuclei, which then assemble into beaded superstructures resembling early protofibrils (Bitan et al., 2003). However, some studies suggest that the presence of Aβ42 pentamers and hexamers may be an experimental artifact; highlighting that Aβ42 oligomerization primarily occurs through dimers and trimers (Pujol-Pina et al., 2015). Furthermore, high-resolution studies of fibril-like interactions in spherical oligomers of Aβ40 have shown the presence of parallel β-sheets, while Aβ42 oligomers lack β-sheet secondary structure, indicating significant differences in the structural characteristics and toxicity of these Aβ forms (Kotler, 2015).

Minor modifications in the Aβ42/Aβ40 ratio have a significant impact on the biophysical and biological characteristics of Aβ mixtures, affecting their aggregation kinetics, the structure of resulting amyloid fibrils, and synaptic functionality both *in vitro* and *in vivo*. A slight increase in the Aβ42/Aβ40 ratio promotes the stabilization of toxic oligomeric forms with intermediate conformations. Initially, these Aβ species have detrimental effects on synapses, leading to toxicity that can spread within cells and ultimately result in neuronal cell death. There is a dynamic balance between toxic and non-toxic intermediates (Kuperstein et al., 2010). Studies have shown that the oligomers formed in the peptide mixture solution are predominantly co-oligomers, especially at the physiological Aβ42 to Aβ40 ratio (1:10), underscoring the significant influence of Aβ40 on oligomer formation and aggregation processes (Meng et al., 2024). While both Aβ40 and Aβ42 initially produce larger oligomers on neurites compared to glass slides, a 1:1 combination of these peptides result in the formation of smaller oligomers attached to neurites than those observed on slides or with either peptide alone. At physiological concentrations, small Aβ oligomers adhere to the membrane and gradually increase in size over time, with the kinetics of this process being influenced by the local ratio of Aβ42 to Aβ40 (Johnson et al., 2013).

Protofibrils formed from a combination of Aβ42 and Aβ40 are mainly composed of Aβ42, with minimal incorporation of Aβ40. However, even in small amounts, Aβ40 can influence the structural characteristics of Aβ42 protofibrils (Terrill-Usery et al., 2016). An increase in the Aβ42/Aβ40 ratio promotes protofibril formation; however, the inclusion of Aβ40 in mixed Aβ solutions significantly hinders this process. When the Aβ42/Aβ40 ratio is elevated, the size of protofibrils remains relatively constant, while the β-sheet structure is strengthened (Terrill-Usery et al., 2016).

Monomeric Aβ40 significantly affects the kinetic stability, solubility, and morphological features of Aβ42 aggregates, hindering their conversion into mature fibrils. When present in approximately equimolar concentrations (Aβ40/Aβ42 around 0.5–1), Aβ40 can inhibit over 50% of fibril formation initiated by monomeric Aβ42. Moreover, the inhibition of protofibrillar Aβ42 fibrillogenesis occurs at lower, substoichiometric ratios (Aβ40/Aβ42 approximately 0.1) (Jan et al., 2008). Recent studies also indicate that Aβ42 aggregation is decelerated by Aβ40, while Aβ40 aggregation is accelerated by Aβ42 in a concentration-dependent manner (Meng et al., 2024).

The Aβ42 fibril is characterized by a single protofilament, whereas the Aβ40 fibril consists of two protofilaments. The three-dimensional configuration of the Aβ42 protofilament is composed of two superimposed, intermolecular, parallel, in-register β-sheets that extend along the axis of the fibril (Lührs et al., 2005). Research employing high-speed AFM to examine the growth process of fibrils indicates that Aβ42 molecules are added alternately to each of the two cross-β subunits within the protofilament (Yagi-Utsumi et al., 2024). Both Aβ42 and Aβ40 fibrils exhibit axial twofold symmetry and have similar protofilament structures. Additionally, the protofilaments of both Aβ40 and Aβ42 fibrils contain the same number of Aβ molecules within each cross-β repeat (Schmidt et al., 2009). Although the fibrillogenesis of both monomeric forms can be initiated by fibrils derived from either peptide, it is the Aβ42 protofibrils that specifically facilitate the fibrillogenesis of monomeric Aβ42, while leaving monomeric Aβ40 unaffected (Jan et al., 2008). Solid-state NMR studies have shown that the C-terminal Ala42, unique to Aβ42, forms a salt bridge with Lys28, serving as a molecular switch for self-recognition and excluding Aβ40 from the process (Xiao et al., 2015).

## 5 Post-translational modifications

The composition of Aβ in the brains of AD patients was analyzed to investigate potential associations between post-translational modifications and the accumulation of this peptide in affected tissues (Roher et al., 1993). Studies have shown that, in addition to the well-known Aβ40/42 variants, Aβ peptides extracted from the brains of AD patients and amyloid plaque cores exhibit truncated N- and C-termini, as well as other post-translational modifications (Kumar et al., 2020; Roher et al., 2017).

The peptide compositions in the cerebrovascular and senile plaque core amyloid deposits of AD patients show significant variability in the amino-terminal region, with a diverse array of peptides starting with each of the initial eleven amino acids in the Aβ sequence and ending with Ala42 (Miller et al., 1993). A physiologically relevant variant of truncated Aβ is generated through initial truncation at the N-terminus at a glutamic acid residue (E3 or E11), which is then cyclized into a pyroglutamate form (pE3 or pE11). Both variants have been detected in elevated levels within the amyloid plaque cores (Scheidt et al., 2024).

The structural characteristics of oligomers and fibrils formed by the peptides pE3-Aβ3-40 and pE11-Aβ11-40 closely resemble those of wild-type Aβ40 (Scheidt et al., 2016; Scheidt et al., 2020; Scheidt et al., 2017). However, pE3-Aβ3-40 shows a higher tendency to adopt β-sheet-dominant structures and form large fibrils in the presence of a helix-stabilizing co-solvent (trifluoroethanol), while wild-type Aβ40 mainly adopts α-helical structures and lacks ThT-positive structures (Dammers et al., 2015). Studies have also suggested that pE11-Aβ11-40 fibrils exhibit greater toxicity compared to wild type Aβ40 and pE3-Aβ3-40 (Scheidt et al., 2017).

Among the truncated N-terminal isoforms, the pE3-Aβ3-42 isoform is the predominant variant, showing a higher β-sheet content and increased aggregation compared to monomeric Aβ42 (Dammers et al., 2017; Nath et al., 2023). This characteristic results in the formation of large fibrils that can be visualized through electron microscopy, a feature absent in Aβ42. Initially, when dissolved, pE3-Aβ3-42 displays two α-helical regions linked by a flexible linker, while the N-terminus remains disordered. Studies suggest that these α-helices act as a transient intermediate stage in the transition to β-sheet and fibril formation of pE3-Aβ3-42 (Dammers et al., 2017). The pE3-Aβ3-42 fibrils exhibit similar positions of β-strands and maintain a conserved turn region surrounding V24, resembling the LS-shaped fibrils of Aβ42 (Gardon et al., 2024).

Phosphorylation of the serine-8 residue (pS8) in Aβ40 facilitates the formation of oligomeric Aβ aggregates, which act as nuclei for fibrillization. This modification increases toxicity, enhances aggregation, accelerates the process, and eliminates the lag phase in aggregation process. Moreover, phosphorylation at serine-8 encourages the development of β-sheet structures (Bagheri and Saboury, 2022; Kumar et al., 2011). The structural characteristics of the N-terminal region in pS8-Aβ40 fibrils display significant differences compared to all previously identified wild-type Aβ40 fibrils, showing strong intra-strand interactions that promote close association between the N terminus and the amyloid core (Hu et al., 2019).

Phosphorylation of serine-8 in Aβ42 enhances β-sheet formation, accelerating amyloid aggregation in a synthetic lipid environment. This modification increases cellular binding affinity and reduces neurotoxic effects (Jamasbi et al., 2017). Despite pS8-Aβ42 aggregating faster than Aβ42, zinc ions inhibit its aggregation. Phosphorylation of Aβ at serine-8, located in the zinc-binding domain, significantly alters zinc-induced oligomerization. Zinc-induced oligomerization of Aβ may serve as a seeding mechanism for neurotoxic Aβ oligomers and aggregates (Barykin et al., 2018).

An additional modification that occurs significantly more frequently in brain samples from AD patients than in healthy controls is Aβ isomerization. For instance, the Aβ1-15 species exhibits around 85% isomerization in insoluble plaques and membrane fractions from the brain samples of patients, while control samples show only 50% (Mukherjee et al., 2021).

The isoAsp7-Aβ variant is the predominant form found in pathological conditions, with Aβ4-X, pGlu3-Aβ, pGlu11-Aβ, and pS8-Aβ following in abundance (Schrempel et al., 2024). Another study suggests that the accumulation of isoD-Aβ starts with aging, while the deposition of pE3-Aβ is more directly linked to AD (Moro et al., 2018). The aggregation of Aβ can be divided into three distinct biochemical stages, characterized by the presence of pE3-Aβ and phosphorylated Aβ in a hierarchical manner. Western blot analysis revealed that in the initial preclinical phase of AD (biochemical stage 1 of Aβ aggregation), various forms of Aβ aggregates—soluble, dispersible, membrane-associated, and plaque-associated—showed no detectable levels of pE3-Aβ or phosphorylated Aβ. In the subsequent biochemical stage 2, pE3-Aβ was detected, while phosphorylated Aβ was exclusively observed in the final stage of Aβ aggregation, which is biochemical stage 3 (Rijal Upadhaya et al., 2014).

In the aggregation assay, isoAsp7-Aβ, pGlu3-Aβ, and pGlu11-Aβ variants showed rapid fibril formation without any delay, while the other variants, which were absent in plaque formations, such as isoAsp27-Aβ, did not exhibit fibril formation (Schrempel et al., 2024). N-terminally modified Aβ variants such as pS8-Aβ40, Y10-nitrated Aβ40 (nY10-Aβ40), and isoAsp7-Aβ40 alter the structural properties and cytotoxicity of wild-type Aβ fibrils through cross-seeding (Hu et al., 2020). For instance, pS8-Aβ40 fibrils can cross-seed with wild-type Aβ40 monomers, resulting in more stable and rigid fibrillar structures compared to self-nucleated wild-type Aβ40 fibrils (Hu et al., 2019).

IsoAsp7-Aβ42 and pS8-Aβ42 isoforms have shown enhanced ability to cross the blood-brain barrier compared to unmodified Aβ42, attributed to distinct endocytosis mechanisms influencing their transport. The lower binding affinity of pS8-Aβ42 and isoAsp7-Aβ42 for RAGE compared to Aβ42 may reduce their intracellular accumulation, facilitating more effective translocation to the abluminal side (Varshavskaya et al., 2024).

## 6 Role of membranes in Aβ aggregation

*In vitro* aggregation studies typically use Aβ concentrations in the micromolar range. However, the physiological levels of Aβ in the brain are in the low nanomolar range, suggesting that spontaneous aggregation of Aβ peptides is unlikely under these conditions (Lyubchenko, 2023). Nonetheless, further research has shown that the spontaneous formation of Aβ oligomers can occur at physiologically relevant concentration. This phenomenon is attributed to a surface aggregation mechanism where the surface acts as a catalyst for aggregation (Lyubchenko, 2020).

### 6.1 Aβ42 aggregation

At nanomolar concentrations, Aβ42 shows a strong preference for surface aggregation over bulk solution pathways (Banerjee et al., 2017). MD simulations indicate that when a monomer interacts with the surface, it undergoes a conformational change. Subsequent binding of another monomer to this altered form leads to the formation of a dimer, causing both monomers to undergo conformational shifts. This surface interaction facilitates the rapid formation of dimers (Banerjee et al., 2017). Energy landscapes of Aβ42 dimerization reveal that disordered states have the lowest energy for Aβ42 monomers, while the lowest energy minima for dimers consist of more ordered structures, primarily β-hairpins. These structures form as Aβ42 folds upon binding to the hydrophobic region of another Aβ42 peptide (Schäffler et al., 2024). Recent experimental studies have shown that the proportion of β-sheets in Aβ increases threefold upon initial interaction with the membrane (Heermant et al., 2025).

**FIGURE 2:**
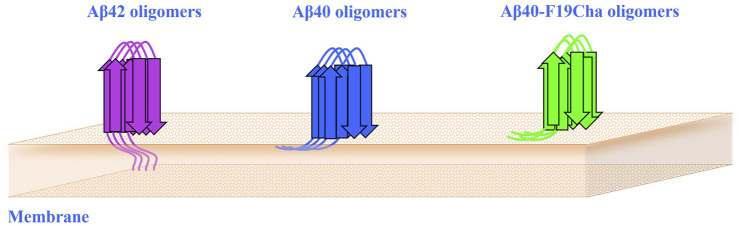
The role of Aβ N-terminal in interaction with membranes. Aβ42’s N-terminus penetrates closer to the lipid bilayer’s center, whereas Aβ40 is located more superficially. The least toxic variant, Aβ40-F19Cha, does not exhibit specific localization.

Distinct differences exist in the interaction behaviours of Aβ42 and Aβ40 monomers when they form dimers. Although the sequence variation between these peptides lies in the C-termini, it is the N-terminal segment that significantly influences their interactions within dimers. The N-terminal region of the Aβ40 peptide contributes to reduced interpeptide interactions, but the additional two residues in Aβ42 counteract this effect (Lv et al., 2013). Furthermore, the N-terminal regions of Aβ42 dimers play a role in Aβ aggregation within membrane environments (Press-Sandler and Miller, 2022). Findings from a single molecule technique (QSLIP) suggest that the N-terminus of Aβ42 penetrates near the core of the lipid bilayer, while the less harmful Aβ40 is located at a shallower level (Figure 2). The least toxic variant, Aβ40-F19Cha, does not exhibit distinct localization (Dey et al., 2024).

Membranes with low packing density promote Aβ42 fibrillation, resulting in shorter fibrils compared to membranes with high packing density. The presence of cis-double bonds in lipid acyl chains reduces packing density, enhancing hydrophobic interactions with Aβ42. Conversely, anionic lipids inhibit Aβ42 fibrillation by establishing strong electrostatic interactions that tightly bind Aβ42 to the membrane surface (Heo et al., 2021). Despite Aβ′s negative charge at physiological pH, studies show that Aβ can interact with anionic lipids through electrostatic forces, involving interactions between positively charged peptide residues and negatively charged lipid head groups (Bokvist et al., 2004; Niu et al., 2018).

Analysis of biomimetic membranes mimicking cellular oxidative stress, compared to mammalian and bacterial membranes, revealed that incorporating oxidized lipids as a cellular stress mimic had effects on peptide self-assembly similar to bacterial mimetic membranes. Electrostatic interactions were found to be crucial in facilitating peptide-membrane binding (John et al., 2023).

All-atom MD simulations have shown that the Aβ42 dodecamer has a more pronounced disruptive effect on neuronal membranes compared to the mature fibril. The study highlights the significance of electrostatic interactions between Aβ and the membrane, with these interactions playing a more crucial role than van der Waals interactions. Additionally, the electrostatic interaction energy associated with fibrils is stronger than that of the dodecamer. The interaction between Aβ and the membrane is mainly influenced by repulsive electrostatic forces between Aβ and the ganglioside GM1 lipid. Importantly, the Aβ42 dodecamer can approach the membrane more closely than the fibril (Nguyen et al., 2022).

MD simulations investigating the impact of cholesterol on the binding of fibrils to lipid bilayers have revealed that Aβ fibrils exhibit a higher affinity for bilayers with elevated cholesterol concentrations. The binding interactions are predominantly governed by electrostatic forces, leading to longer lifetimes and increased binding frequency as cholesterol levels increase (Dias et al., 2020).

### 6.2 Aβ40 aggregation

**FIGURE 3:**
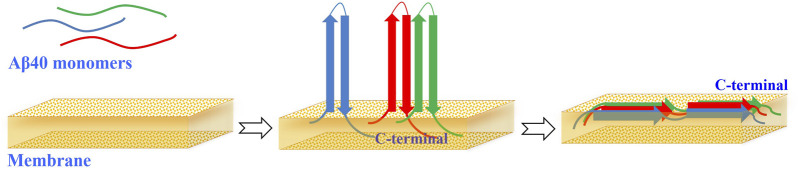
Aβ40 changes in the presence of membranes. When unbound, Aβ40 adopts a random coil configuration. However, when bound to membranes, Aβ40 oligomers form an antiparallel β-sheet structure, with the C-terminus positioned deeper than the N-terminus. The integration of Aβ40 into membrane promotes the transition from antiparallel to parallel β-sheet arrangements.

The CD spectrum of unbound Aβ40 indicates a random coil structure (Figure 3). Aβ40 monomers do not immediately change their conformation upon interacting with membranes, remaining mostly unfolded with some minor conformations. However, as fibrillation commences, Aβ40 progressively transitions from its native random coil structure to a β-sheet conformation (Bera et al., 2020).

Experiments using fluorescence and Raman spectroscopy on membrane-bound Aβ40 oligomers have shown that these structures adopt an antiparallel β-sheet conformation, with the C-terminus more deeply embedded than the N-terminus (Bhowmik et al., 2015; Chandra et al., 2018). The integration of Aβ40 into membrane structures promotes the shift from antiparallel to parallel β-sheet configurations (Figure 3), which occurs abruptly after a delay, indicating a cooperative mechanism at play (Heermant et al., 2025). This transition is influenced by the charge of the lipids, with negatively charged membrane interfaces amplifying the observed effects. In contrast, Aβ behaves similarly in the presence of neutral lipids, but to a lesser extent. Notably, in positively charged lipid membranes, there are no detectable amide I signals characteristic of β-sheets (Heermant et al., 2025).

Various membrane compositions have been shown to enhance the aggregation kinetics of Aβ40, accompanied by distinct transitions in the peptide’s secondary structure. The core and C-terminal residues primarily influence the interactions between Aβ40 and the membrane. These conformers of Aβ40, which exhibit structural differences, are morphologically and functionally different from Aβ40 that lacks lipid components (Bera et al., 2020). MD simulations indicate that the secondary structure of the C-sheet remains largely intact within the membrane, whereas the N-sheet exhibits fraying at its ends. After the formation of a small oligomeric seed within the membranes, it is subsequently released into the surrounding aqueous environment. The residue Y10 serves to anchor the N-sheet to the membranes, while the C-sheet is liberated (Kunnath Muhammedkutty and Zhou, 2025).

## 7 Membrane damage

At low nanomolar concentrations, Aβ42 has been shown to have no harmful effects on supported lipid bilayers (van Deventer and Lyubchenko, 2024). Previous studies using FRET microscopy have similarly indicated that the membrane model remains mostly intact at a concentration of 100 nM Aβ (Chandra et al., 2018). Additionally, initial experiments on calcium leakage showed that treatment with Aβ at concentrations below 10 nM did not lead to significant toxicity compared to the control group (Johnson et al., 2013). This raises questions about the mechanisms through which Aβ may cause membrane damage.

At concentrations below 10 nM, monomers consistently bind to the membrane surface without forming oligomers or disrupting the membrane. As the concentration increases from 10 nM to several hundred nanomolar, monomers continue to bind uniformly while dimers and small oligomers start to appear. Dimers do not cause membrane permeabilization, but larger oligomers can induce permeabilization, with each oligomer contributing to ion conductance of less than 10 pS per pore (Schauerte et al., 2010).

The disruption of membranes is associated with amyloid species that exist as transient small oligomeric entities during the initial phases of aggregation, characterized by morphologies comprising small globular species measuring less than 10 nm in diameter (Feuillie et al., 2020). Additionally, other research indicates that the detrimental effects of Aβ42, with an average diameter of 5.2 nm, are significantly more pronounced than those associated with the smaller 3.9 nm species (Yasumoto et al., 2019). Moreover, large soluble oligomers of Aβ (≥150 kDa) from AD brains are much less toxic than the smaller oligomers (∼8–70 kDa) they break down into (Yang et al., 2017). AFM imaging revealed that Aβ deposits gradually separated from the membrane, leading to the excision of portions of the underlying bilayer (Azouz et al., 2019). Furthermore, time-lapse AFM imaging in solution demonstrates that, as time progresses, both the size and number of oligomers increase while they are released into the solution. This finding suggests that the aggregates formed could serve as nucleation sites, promoting additional aggregation within the surrounding solution (Banerjee et al., 2017).

**FIGURE 4:**
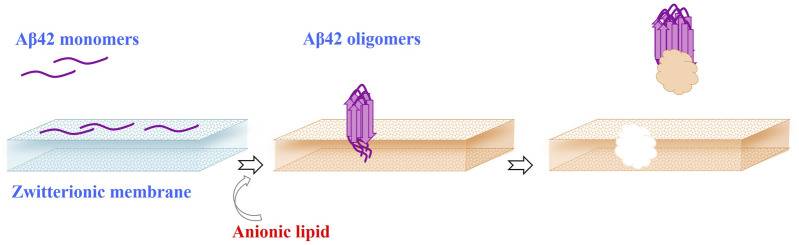
Interaction of Aβ42 with membrane. Aβ42 oligomers exhibit a higher propensity to penetrate the membrane and form pores, in contrast to monomers that simply bind to the outer surface of the membrane without penetrating it. Aβ42 monomers show limited adsorption to zwitterionic membranes, failing to form aggregates. However, the presence of anionic phospholipids in the membrane induces the oligomerization of Aβ42 monomers, leading to membrane disruption.

Aβ42 oligomers have a greater tendency to infiltrate and create perforations in the membrane, unlike monomers, which only adhere to the membrane’s exterior without the ability to penetrate its structure (Figure 4) (Nag et al., 2013; Robinson et al., 2025; Sarkar et al., 2013). Furthermore, Aβ42 oligomers exhibit significant cytotoxic effects and are easily internalized by neurons, whereas Aβ42 fibrils show reduced internalization and lack any toxic effects. Additionally, sonication of Aβ42 fibrils produces species similar in size to oligomers, yet these remain non-toxic. Therefore, it is clear that Aβ42 oligomers possess distinct characteristics that contribute to their neurotoxic capabilities (Vadukul et al., 2020). The greater bilayer perturbations caused by G37C oligomers compared to Aβ42 confirm this observation, suggesting that oligomers unable to progress to the fibril state can exhibit significant toxicity (Azouz et al., 2019).

Numerous studies using AFM methods, electrophysiology, and cell biology have shown that Aβ can induce pore-like structures and stimulate channel activity in membranes (Connelly et al., 2012; Demuro et al., 2011; Lal et al., 2007; Quist et al., 2005). Membrane patches from HEK293 cells were used to investigate the channel-forming abilities of monomeric, oligomeric, and fibrillar forms of Aβ40 and Aβ42 in a more physiological context. The results revealed that Aβ42 oligomers form voltage-independent, non-selective ion channels, while Aβ40 oligomers, fibers, and monomers do not exhibit channel formation (Bode et al., 2017). A minimum of six monomeric subunits of Aβ40 oligomers is required to induce significant conductance in model membranes, but the levels of these oligomeric structures were found to be minimal, suggesting minor effects on neuronal function (Johnson et al., 2013).

High-speed AFM studies showed that stable oligomeric species like Aβ42-oG37C, which do not fibrillate, do not interact with membranes composed of phosphatidylcholine and sphingomyelin. The presence of ganglioside GM1 is crucial for the peptide’s interaction with the membrane, facilitating its insertion through nearby cholesterol and leading to membrane disruption (Ewald et al., 2019). Simulations highlighted the roles of GM1 and cholesterol in stabilizing membrane-embedded β-sheets, as well as functions of Y10 and K28 in releasing small oligomeric seeds into the solution (Kunnath Muhammedkutty and Zhou, 2025). MD simulations have shown that sphingomyelin, unlike GM1, promotes a β-sheet-rich conformation in Aβ42 monomers at physiologically relevant concentrations. The increased rigidity of the bilayer due to sphingomyelin reduces interactions with the N-terminus of Aβ42, inhibiting its embedding and promoting a β-sheet structure in the N-terminal region, resembling higher-order Aβ fibrils (Owen et al., 2018).

Calorimetric investigations revealed that Aβ42 fibrils, oligomers, and monomers can bind to or integrate into bilayers in a liquid-ordered state, regardless of their electric charge. However, monomers do not interact with electrically neutral bilayers (Ahyayauch et al., 2024). The key factor influencing the aggregation process is the affinity of Aβ monomers for the membrane surface, rather than the concentration of Aβ (Lyubchenko, 2020). Monomeric Aβ42 weakly adsorbs to zwitterionic DOPC membranes without forming aggregates. However, the addition of 10 mol% DOPS (anionic phospholipids) to the outer leaflet of the membrane induces the oligomerization of Aβ42 monomers (Figure 4), leading to membrane damage (Robinson et al., 2023). AFM results suggest that the inclusion of cholesterol or GM1 at a concentration of 10 mol% in zwitterionic POPC membranes promotes the formation of a liquid-disordered-phase domain, facilitating Aβ42 aggregation and causing membrane fragmentation (Azouz et al., 2019).

Studies on POPC membranes with cholesterol or GM1 have shown that Aβ42 induces the formation of small aggregates on cholesterol-containing membranes, measuring several nanometers, and small fibers on GM1-containing membranes, approximately 10 nm in height. Detachment of these deposits from POPC membranes with cholesterol creates perforations in the outer layer, while detachment from POPC membranes with GM1 results in holes within the bilayer (Azouz et al., 2019). Additionally, membrane thickness plays a significant role in the morphological changes upon Aβ40 adsorption. Thicker DOPC and POPC supported lipid bilayers undergo remodeling, forming elongated tubules and globular structures to alleviate membrane stress in response to varying Aβ40 concentrations. In contrast, thin DLPC membranes do not undergo significant membrane remodeling (Meker et al., 2018).

## 8 Membrane changes in AD and aging

Numerous studies have shown that Aβ, when externally introduced, selectively binds to specific cells within a seemingly uniform cell population in culture. The binding affinity of Aβ to cellular membranes is significantly influenced by the presence of specific lipid constituents like cholesterol, sphingolipids, gangliosides, and various phospholipids (Burke et al., 2013). The structure of cellular membranes undergoes changes with aging and in AD (Drolle et al., 2017). Analysis of the lipid composition of lipid rafts from both control and early-stage AD subjects suggests that lipid composition modifications within cortical lipid rafts occur early in sporadic AD, facilitating interactions between APP and BACE1. These lipid changes in AD lipid rafts result in increased membrane order and viscosity in these regions (Fabelo et al., 2014). Research utilizing various techniques, has shown that multicomponent lipid models, mimicking both healthy and pathological neuronal membrane states, exhibit distinct variations in their nanoscale architecture and physical properties. Moreover, these models demonstrate different interactions with Aβ42, indicating that those representing diseased membranes are more prone to interactions with Aβ and its detrimental effects (Drolle et al., 2017).

## 9 Role of copper in Aβ accumulation and membrane damage

Copper significantly influences the aggregation of Aβ42, enhancing its cytotoxicity while having minimal impact on Aβ40. This is attributed to the strong interactions between copper and Aβ42, leading to conformational changes and stabilization of toxic nanoscale oligomers, unlike the effect on Aβ40 (Bagheri et al., 2022; Jin et al., 2011). The Arctic variant of Aβ40 exhibits unique behavior compared to wild-type Aβ40, as substoichiometric copper concentrations can induce the formation of toxic oligomers similar to those formed with wild-type Aβ42 (Tian et al., 2024). Copper also affects the assembly of Aβ40 and Aβ42 differently, promoting fibril formation of Aβ40 by enhancing primary nucleation, while stabilizing Aβ42 as prefibrillar oligomers and protofibrils. Notably, the introduction of copper to pre-existing Aβ42 fibrils leads to their disassembly, reverting them to protofibrils and oligomers (Tian et al., 2024).

Furthermore, copper overload leads to increased cholesterol synthesis through ROS-dependent and independent pathways, elevating cholesterol levels in cell membranes and lipid rafts. Despite unchanged total APP levels, its presence in lipid rafts increases during copper overload, correlating with higher Aβ concentrations in the culture medium (Bagheri et al., 2018; Zubillaga et al., 2022).

## 10 Concluding remarks

This article aims to explore the differences in the accumulation of Aβ40 and Aβ42 from a biophysical perspective, drawing on recent scientific advancements. It also discusses the detrimental effects these amyloids can have on membranes, providing a comprehensive overview for readers interested in this topic.

In summary, despite differing in only two amino acids, Aβ40 and Aβ42 exhibit significant structural and functional differences. Aβ42 has a higher propensity to form fibrils, but it also produces oligomers with distinct structures from fibrils, leading to its toxic effects. Conversely, Aβ40 oligomers tend to adopt a linear configuration when exceeding tetramers, but their specific structure, particularly the dynamics of the C-terminal region, reducing their fibril-forming tendency. The structural disparities of beta amyloids influence their interactions with membranes, with the toxic effects on membranes primarily attributed to the oligomers of Aβ42. Aging and AD-related changes in membranes make them vulnerable to damage induced by amyloid. Additionally, disruption of copper homeostasis increases cholesterol and amyloid production, and stabilizes toxic oligomeric species that exacerbate membrane damage. The efficacy of medications that stabilize small, harmless aggregates, preventing the formation of larger toxic species, supports the toxic oligomer hypothesis.
